# A practical method to estimate the benefits of improved road network reliability: an application to departing air passengers

**DOI:** 10.1007/s11116-017-9764-4

**Published:** 2017-03-09

**Authors:** Eric Kroes, Paul Koster, Stefanie Peer

**Affiliations:** 1 Significance, Koninginnnegracht 23, 2514 AB The Hague, The Netherlands; 2 Department of Spatial Economics, VU University Amsterdam, De Boelelaan 1105, 1081 HV Amsterdam, The Netherlands; 3 Tinbergen Institute, Gustav Mahlerplein 117, 1082 MS Amsterdam, The Netherlands; 4 Vienna University of Economics and Business, Welthandelsplatz 1, 1020 Vienna, Austria

**Keywords:** Value of reliability, Airport access, Accessibility, Travel time variability

## Abstract

This paper develops and applies a practical method to estimate the benefits of improved reliability of road networks. We present a general methodology to estimate the scheduling costs due to travel time variability for car travel. In contrast to existing practical methods, we explicitly consider the effect of travel time variability on departure time choices. We focus on situations when only mean delays are known, which is typically the case when standard transport models are used. We first show how travel time variability can be predicted from mean delays. We then estimate the scheduling costs of travellers, taking into account their optimal departure time choice given the estimated travel time variability. We illustrate the methodology for air passengers traveling by car to Amsterdam Schiphol Airport. We find that on average planned improvements in network reliability only lead to a small reduction in access costs per trip in absolute terms, mainly because most air passengers drive to the airport outside peak hours, when travel time variability tends to be low. However, in relative terms the reduction in access costs due to the improvements in network reliability is substantial. In our case we find that for every 1 Euro reduction in travel time costs, there is an additional cost reduction of 0.7 Euro due to lower travel time variability, and hence lower scheduling costs. Ignoring the benefits from improved reliability may therefore lead to a severe underestimation of the total benefits of infrastructure improvements.

## Introduction

Past studies have shown that the economic benefits from more reliable travel times are substantial, as they usually amount to 10–25% of the benefits associated with shorter travel times (e.g. Fosgerau et al. 2008; Eliasson 2006; Peer et al. 2012). The results of transport project appraisals may thus be significantly underestimated if travel time variability is not considered. In recent years various countries such as the US, the UK, the Netherlands and Sweden have introduced guidelines on how to include travel time variability in appraisals of transport projects (see De Jong and Bliemer 2015, for an overview). The proposed methods with the aim to quantify the costs associated with travel time variability differ in terms of their complexity as well as feasibility; the latter mainly being determined by the limitations of existing transport models. This paper suggests a practical method that uses standard output of transport models, but yet models the underlying scheduling decisions of travellers in more realistic ways than existing models.

De Jong and Bliemer (2015) suggest to categorize the available methods used to quantify the costs of travel time variability according to four criteria:

1. Are network users assumed to take into account travel time variability in their travel choices (e.g. concerning departure time, route, mode or destination)?
2. Is (the response mechanism to) travel time variability formulated in terms of a simple measure of dispersion (usually the standard deviation), or is it expressed in terms of schedule delays with respect to the preferred arrival time? The latter requires the presence of a departure time model.
3. Is the relationship between travel times and travel time variability exogenous (e.g. empirically estimated), or endogenous (e.g. generated via Monte Carlo simulations)?
4. Does the method consider only a single “average weekday”, or does it distinguish between different scenarios (e.g. different weekdays, different weather conditions, holidays)?

Based on these four criteria, De Jong and Bliemer (2015) derive three different methods, which differ in their overall complexity, with Method 1 being the simplest and hence most feasible method, Method 2 being of intermediate complexity, and Method 3 representing the most complex and ideal model setup, which currently cannot yet be introduced at a wider scale. Method 1 corresponds to a “post-processing module”, which uses the output of standard transport models. It assumes that travellers do not take into account travel time variability in their travel choices, and it represents travel time variability in terms of a simple dispersion measure. The relationship between travel times and the dispersion measure is exogenously given, and usually assumed to be linear (“reliability ratio approach”). Moreover, it typically only considers one scenario. Methods 2 and 3 relax these restrictions, with the main difference between these two being the definition of variability in terms of schedule delays and the endogenous relationship between travel times and variability, which are both only introduced in Method 3.

In this paper, we develop a method to quantify the costs of travel time variability that essentially still corresponds to Method 1, as it undoubtedly is a post-processing module using standard output of transport models.^1^ However, it extends existing approaches that belong to Method 1 by adding various characteristics of Methods 2 and 3.

Most importantly, we define a reduced-form function of expected travel costs that defines travel time variability in terms of schedule delays,^2^ which De Jong and Bliemer (2015) see as a characteristic of the most ideal and advanced method. Our cost function thus takes into account explicitly the travellers’ trip timing decisions: travellers are assumed to schedule their trips in such a way that they trade off the costs associated with arriving early at their destination against the costs of arriving late. Everything else equal, in our model travellers will leave earlier from home if travel times become more unreliable.^3^ Our modeling approach allows us to include a discrete penalty for lateness in the expected travel cost function (which is particularly relevant in our application). Moreover, it allows us to combine it with the output of a standard transport model: mean travel times. If we had followed Fosgerau and Karlström (2010)—who showed that the scheduling approach is (under certain conditions) theoretically equivalent to the so-called reliability ratio approach, which is based on the assumption of a linear relation between the costs of travel time variability and the standard deviation of the delays—we would also have needed to predict the mean lateness factor.

Although we assume that the relation between travel times and travel time variability is exogenous (see criterion 3 in the list above), we introduce two features that go beyond most existing approaches to quantify the costs of travel time variability. First, we assume that the travel time distributions are log-normal rather than normal (as assumed in most existing models), implying that we are able to approximate the right-skewed nature of the distributions that are frequently observed in empirical analysis of travel time data [see for example Rakha et al. (2010) and Emam and Ai-Deek (2006)]. Using a log-normal distribution allows us as well to compare the costs of travel time variability across different scenarios without assuming that the standardized distributions are the same before and after the change in the transport network. Second, we assume that the relationship between mean delay and variability is origin–destination (OD)-pair-specific and dependent on the time of the day. The empirically calibrated relationships are based on the work of Kouwenhoven and Warffemius (2015). Their research has confirmed a strong positive correlation between mean delay and travel time variability, which has also been identified in an earlier study by Peer et al. (2012).

While the method developed in this paper is generic in its nature, we illustrate how it can be applied to measure the costs due to unreliable travel times for Dutch car travellers going to Amsterdam Schiphol Airport to travel by plane from there. In 2013, about 40% of the travellers to Amsterdam Schiphol Airport accessed the airport by car (taxi travel excluded). This is a situation in which travellers incur potentially large costs of unreliability, particularly when they miss their flight. As a consequence, most use a safety margin (buffer time), which is the additional time that the travellers leave earlier from home due to delays. The intuitive behavioural response that this buffer becomes larger as travel time variability increases was already suggested more than 45 years ago by Thomson (1968), Gaver (1968) and Knight (1974). As far as we are aware, Hall (1983) was the first author to apply this principle to departure time choices of air passengers travelling to the airport. Koster et al. (2011) adapted the linear scheduling model with random travel times of Noland and Small (1995) to air passengers driving to the airport, by adding a penalty for missing a flight.

In our Amsterdam Schiphol Airport case study, we compare the airport access costs of car travellers arising from mean delays and travel time unreliability for two different network specifications: (1) the Dutch road network as it existed in 2010, and (2) an improved version of that network as it is planned for 2020. The 2020 road network benefits from considerable investments in additional road capacity, which are expected to decrease mean delays and to induce higher reliability. By analysing the differences in access costs between these two networks we learn how the reliability benefits of the road improvement program add to the more traditional benefits due to decreases in mean travel time.

The paper proceeds as follows. “Behavioural scheduling responses to travel time variability” section introduces the methodology, specifically the reduced-form function of expected travel costs. “Parametrization” section discusses the assumptions that will be made in the application of the method to air passengers accessing Amsterdam Schiphol Airport by car concerning scheduling preferences as well as the relation between mean delay and travel time variability. “Application: accessing Amsterdam Schiphol Airport by car” section describes the background of the application and reports the numerical results. Finally, “Conclusions” section concludes.

## Behavioural scheduling responses to travel time variability

We first introduce the expected travel cost function that we use in our model. It is based on the work of Noland and Small (1995), who extended the standard scheduling model of Vickrey (1969) to allow for randomness in travel times. More specifically, we employ a specification that has been introduced by Koster et al. (2011) to model expected airport access costs, as we will apply our model in the context of air passengers travelling to the airport.^4^ In their model, travellers minimize their expected access costs to the airport, ${\mathbb {E}}(C(H))$ by deciding on the optimal safety margin *H* when departing from home. They take into account this safety margin because travel times may exceed free-flow travel time. For notational reasons, we assume that travel times consist of a fixed free-flow travel time $T_{f}$, and a variable delay *D*. *H* is then defined as the additional minutes that a traveller leaves earlier from home due to delays. When there is no variability in delays, the optimal safety margin is equal to the mean delay $\mu$.

The expected access costs are then a function of the free-flow travel time $T_{f}$, the expected delay $\mathbb {E}(D)$, as well as the (expected) schedule delays early and late (denoted by *SDE* and *SDL*), which are the costs associated with arriving earlier or later than the (exogenously given) preferred arrival time at the airport. The (exogenous) preferred arrival time pins down $T_{Airport}$, which is the (exogenously given) final check-in time of the traveller minus the preferred arrival time for a given scheduled flight time. It can thus be interpreted as the time that travellers prefer to spend at the airport before the final check-in time.

Since travellers may miss their flight if they arrive at the airport too late, a corresponding penalty term (specified as the percentage probability of missing a flight $PMF(H,T_{Airport})$) is included in the cost function. When $T_{Airport}$ equals 0, this penalty term is similar to the additional discrete lateness penalty $\theta$ proposed by Small (1982). Note that for our analysis it is assumed that flights are not delayed and that travellers do not adjust their preferred arrival time when the travel time distribution changes.

We further assume that delays follow a two-parameter log-normal distribution: $\ln (D)\sim \mathbb {N}(\tau ,\kappa )$, where $\tau$ is the mean and $\kappa$ is the standard deviation of the underlying normal distribution. The probability density function is further denoted by $f_{logn}(D)$, and the cumulative probability distribution by $F_{logn}(D)$). The shape of the distribution is assumed to be independent of the time of the day. The parameters $\tau$ and $\kappa$, i.e. the shape and scale parameter of the distribution, can be derived analytically if the expected delay $\mathbb {E}(D))$ (equal to the mean delay $\mu$) and the *estimated* standard deviation ${\hat{\sigma }}$ of the travel time distribution are known (we use a hat to indicate that the standard deviation is estimated):

1 $$\begin{aligned} \tau =\log (\mu )-\frac{1}{2}\log \left( 1+\frac{{\hat{\sigma }}^{2}}{\mu ^{2}}\right) \quad \text { and } \quad\kappa =\sqrt{\log \left( 1+\frac{{\hat{\sigma }}^{2}}{\mu ^{2}}\right) }, \end{aligned}$$

This implies that the log-normal distribution of delays is fully determined by the mean delay $\mu$ (which is a standard output of network models) and the standard deviation (which we will estimate using the mean delays as inputs; see “Travel time distributions” section). Note that a similar approach can be applied to other (travel delay) distributions that are defined by two parameters.

The expected schedule delays ($\mathbb {E}(SDE;H)$ and $\mathbb {E}(SDL;H)$) are defined as follows. For a given delay *D* and safety margin *H*, the schedule delay early is defined as $SDE=\max (0,H-D)$, and schedule delay late is defined as $SDL=\max (0,D-H)$. To derive the expected schedule delay early, $\mathbb {E}(SDE;H)$, we take a probability weighted average over early arrivals by integrating over all possible early arrivals. Because delays are assumed to be positive (hence, travel times can by definition not be shorter than the free-flow travel time), the integral starts at $D=0$. And it ends at $D=H$, because then a traveller arrives exactly on time, and the schedule delay early will be 0. Substituting $f_{logn}(D)=\frac{1}{D\kappa \sqrt{2\pi }}\exp (-\frac{(\log (D)-\tau )^{2}}{2\kappa ^{2}})$ gives^5^:

2 $$\begin{aligned} \mathbb {E}(SDE;H)=\int _{0}^{H}(H-D)f_{logn}(D)dD=H F_{logn}(H)-\mu F_{logn}\left( \frac{H}{\exp (\kappa ^{2})}\right) \end{aligned}$$

Similarly, the expected schedule delay late $\mathbb {E}(SDL;H)$ can be derived by integrating over all late arrivals. The integral starts at $D=0$, and ends at infinity. Substituting $f_{logn}(D)$ gives^6^:

3 $$\begin{aligned} \mathbb {E}(SDL;H)=\int _{H}^{\infty }(D-H)f_{logn}(D)dD=H F_{logn}(H)-\mu F_{logn}\left( \frac{H}{\exp (\kappa ^{2})}\right) +(\mu -H). \end{aligned}$$

The final component of the expected access cost function is the percentage probability of missing a flight $PMF(H,T_{Airport})$. It depends on the planned time spent at the airport, $T_{airport}$. Travellers who prefer to spend more time at the airport have a lower probability of missing the flight. Therefore, $T_{Airport}$ includes the behavioural response to airport service time delay, which is assumed to be unrelated to delays on the road:

4 $$\begin{aligned} PMF(H,T_{Airport})=100\int _{H+T_{airport}}^{\infty }f_{logn}(D)dD=100(1-F_{logn}(H+T_{airport})). \end{aligned}$$

Finally, combining Eqs. 2, 3 and 4, the expected access cost function can be written as follows:

5 $$\begin{aligned} \mathbb {E}(C(H)) & =   \alpha (T_{f}+\mathbb {E}(D))+\beta \mathbb {E}(SDE;H)+\gamma \mathbb {E}(SDL;H)+\theta PMF(H,T_{Airport})\nonumber \\ & =   \alpha (T_{f}+\mu )+(\beta +\gamma )\left( H F_{logn}(H)-\mu F_{logn}\left( \frac{H}{\exp (\kappa ^{2})}\right) \right) +\gamma (\mu -H)+\theta 100(1-F_{logn}(H+T_{airport})) \end{aligned}$$

where $T_f$ is the free flow travel time, $\alpha$ is the value of access time, $\beta$ is the value of schedule delay early, $\gamma$ is the value of schedule delay late, and $\theta$ is the value of the percentage probability to miss a flight.

Travellers optimize this expected access cost function and choose their optimal safety margin $H^{*}$, resulting in minimal expected access costs $\mathbb {E}(C(H^{*}))$. There is no closed-form solution available for $\mathbb {E}(C(H^{*}))$. Therefore, we determine $H^{*}$ and $\mathbb {E}(C(H^{*}))$ numerically, using a behaviourally plausible step-size for *H* of 5 min.^7^

## Parametrization

### Preferences

For the preference parameters that enter the airport access cost function (Eq. 5), we use the median of the panel mixed logit estimates of Koster et al. (2011), which are based on a stated preference survey among 345 business and 625 non-business travellers (also our empirical analysis distinguishes between business and non-business travellers). The assumptions concerning the preferences are summarized in Table 1. Not surprisingly, business travellers have higher willingness to pay values than non-business travellers, and have a preferred arrival time closer to the final check-in time, meaning that they spend on average less time at the airport. Moreover, the specification of Koster et al. (2011) allows for a proportional difference in preference parameters between business and non-business travellers, which results from a difference in the marginal utility of income: at average income levels, the assumed values for $\alpha , \beta , \gamma$ and $\theta$ of business travellers are 37% higher than the values for non-business travellers.

**Table 1:** Assumed values for the preference parameters

	Business	Non-business
$\alpha$	39.71	28.93
$\beta$	32.19	23.45
$\gamma$	47.07	34.29
$\theta$	8.51	6.20
$$T_{Airport} $$	1.19	1.46

Values for $\alpha ,\beta$ and $\gamma$ are in €/*h*, whereas the value for $\theta$ is in €/%. The value for $T_{Airport}$ is in hours

### Travel time distributions

As shown in “Behavioural scheduling responses to travel time variability” section, the expected access costs to the airport depend on the standard deviation of the travel time distributions. However, standard transport models are typically not able to generate travel time distributions, as they usually only provide estimates of the mean delay, implying that the standard deviation can only be derived using additional assumptions. We base the prediction of the standard deviations on prior research by Peer et al. (2012) and Kouwenhoven and Warffemius (2015), who both find a strong positive correlation between the mean delay and the standard deviation of the delays using travel time data from Dutch motorways. Specifically, we will apply the functional forms derived and empirically validated by Kouwenhoven and Warffemius (2015). They use travel time data from 250 Dutch highway routes^8^ to determine the relationship between travel time variability (represented by the standard deviation ${\hat{\sigma }}$)^9^ and mean delay $\mu$. Their preferred functional form also controls for distance *L*, rendering the relationship route-specific:

6 $$\begin{aligned} {\hat{\sigma }}=a_1+a_2\mu +a_3 \log _{10}(\mu +1)+a_4 L, \end{aligned}$$

**Fig. 1:**
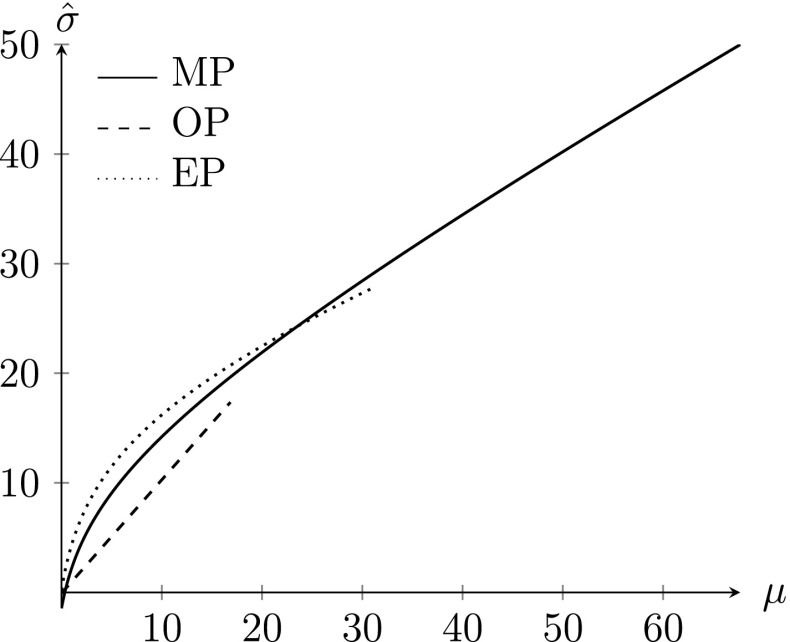
Estimated standard deviation for D = 100. *Note* functions are plotted on the domain of $\mu$

Kouwenhoven and Warffemius (2015) estimate three separate models for observations during the morning peak, the mid-day period and the evening peak. They find that the estimated coefficients differ significantly across the three time periods.^10^ Table 2 reports these coefficients, and Fig. 1 plots the predicted standard deviation as a function of mean delay $\mu$ for the three time periods and a distance *L* of 100 km.^11^

**Table 2:** Coefficients for the empirical relationship between the standard deviation and mean delay based on Kouwenhoven and Warffemius (2015)

	Time period			Units
	Morning peak (MP)	Off-peak (OP)	Evening peak (EP)	
$a_1$	−0.540	−0.066	−0.901	min
$a_2$	0.476	1.034	0.268	–
$a_3$	4.538	–	5.555	min
$a_4$	−0.009	–	0.011	min/km

For the mid-day period, $a_3$ and $a_4$ were found insignificant

## Application: accessing Amsterdam Schiphol Airport by car

### Implementation using a large scale transport model

For our practical case analysis, we use the Dutch National Transport Model System [NMS, see for instance: Gunn (1994)] to predict mean delays for trips with the destination Amsterdam Schiphol Airport (AMS). The NMS is a large, comprehensive transport model system that is based on discrete choice models for trip frequency, destination choice, mode choice, and time-of-day choice. It is the ‘standard’ tool, developed and used since 1985 in the Netherlands, for assessing the effects of transport policies. The model distinguishes 1379 origin and destination zones, so it allows for a highly detailed spatial analysis of the accessibility of Amsterdam Schiphol Airport from all regions in the Netherlands. Additionally, the model differentiates between three time periods: the morning peak (MP) which lasts from 7:00 to 9:00, the evening peak (EP) which starts at 16:00 and ends at 18:00, and the remaining hours of the day (OP), for an “average working day”. Therefore, the model provides estimates for the mean travel time delay for each of the origin zones and for each of these three periods separately.

The NMS uses a highly disaggregate population data base and simulates demand for six different modes of transport, while distinguishing ten different travel purposes. The resulting origin–destination flows are assigned to the road network using Qblok, an equilibrium type car traffic assignment model that takes account of input flow restrictions due to congestion effects upstream (Bakker et al. 1994). Furthermore, it uses speed-flow curve information calibrated on data of the Dutch motorway network. As usual, link travel times are equal to their free flow travel time plus an estimated amount of delay, where mean delay depends on the volume/capacity ratio. The NMS road network represents the entire road network of the Netherlands, including urban roads, provincial roads and motorways. All zones are connected to urban and/or provincial roads only. On average, some 65% of the distances are travelled on motorways.

We apply our model to two different situations. First, the base year car traffic OD matrix of 2010 is assigned to the road network that was available in year 2010. Second, the same car traffic OD matrix of 2010 is assigned to an improved road network for the year 2020. The 2020 network contains all the infrastructure improvements that have been planned and anticipated for that year. This enables us to establish the effects of road network improvements on mean travel times, and hence the expected access costs.

We assume that the overall number of air travellers arriving by car to the airport does not change between 2010 and 2020, hence demand is assumed to be constant. Developments between 2010 and 2020, and improvements in access costs are therefore assumed not to lead to additional car trips to Amsterdam Schiphol Airport. Although this is not a realistic assumption, it makes the interpretation of the results easier, because all changes in travel time distributions are due to network effects only. The numbers of passengers arriving by car at Schiphol in 2010 as included in the model have been derived from large-scale air passenger counts and surveys conducted at the airport, the so-called ‘continuous Schiphol-survey’. This survey has been carried out since many years. About 60.000 departing air passengers per year are interviewed resulting in accurate data about their travel and personal characteristics. A stratified sample and expansion procedure is applied to ensure that all air destinations and nationalities of passengers are included in the survey.

Our analysis concerns an “average working day”. According to the survey results in total about 8.3 million air passengers travelled to Amsterdam Schiphol Airport on all working days of 2010, implying on average 26,000 travellers per working day.^12^ Table 2 shows a breakdown of these travellers by type of travel purpose and by time of the day. The groups of business and non-business travellers do not differ much in size. They account for 52 and 48% of travellers, respectively. Interestingly, most passengers appear to travel to the airport outside the peak periods. As expected, non-business travellers are more likely to travel to the airport outside peak hours than business travellers.

**Table 3:** Daily number of travellers going to Schiphol Airport based on NMS 2010

	Morning peak (MP)	Off-peak (OP)	Evening peak (EP)	Total
Business	2172	9095	2154	13,422
Non-business	1825	9348	1242	12,414
Total	3997	18,443	3396	25,836

### Numerical results

#### Introduction

This subsection discusses the numerical results. We compare the results for the Dutch road network of 2010 with those for the road network of 2020. For 2020, substantial infrastructure investments will have been made to alleviate congestion at the key bottlenecks in the network. These investments have an impact on the travel time distribution of every OD-pair, and therefore result in travel time and travel time reliability gains for departing air travellers who travel by car. We first provide a numerical example for one OD-pair in order to show how the model works (“Introduction” section). The analysis is then repeated for all 1377 origins in the analysis, and the aggregate results will be presented in “Results for the entire road network” section.

#### Example for one OD-pair

**Fig. 2:**
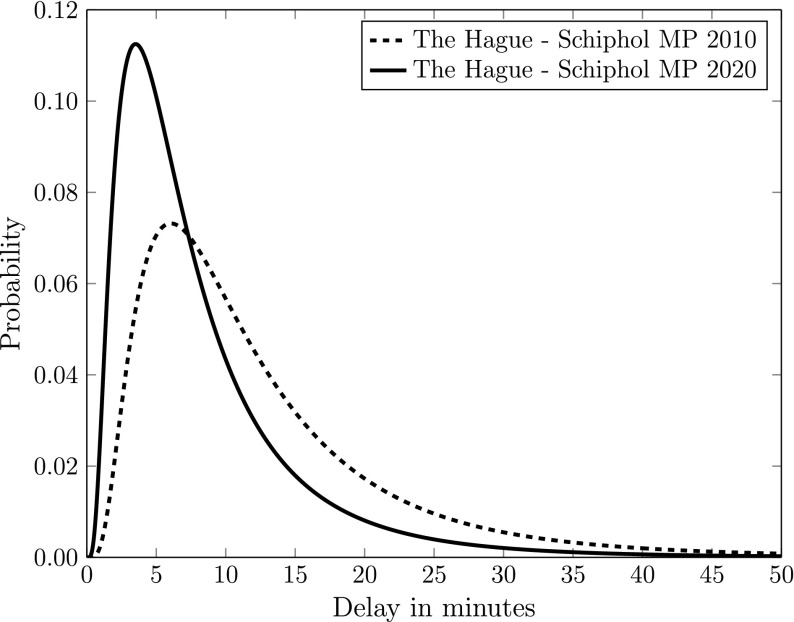
Travel time distributions for the 2010 and 2020 road networks for one OD-pair (The Hague–Amsterdam Schiphol Airport, morning peak)

To illustrate how the model works, we select one specific OD-pair, where the origin is in the city centre of The Hague, and the destination is Schiphol Airport. The NMS provides estimates of the mean delay for 2010 (12.7 min) and 2020 (8.3 min) for this OD-pair. Using the prediction model of “Parametrization” section, we obtain travel time distributions for 2010 and for 2020 for the morning peak, the evening peak and the rest of the day (off-peak). Figure 2 gives the travel time distributions for the morning peak.

This figure clearly shows the change in the travel delay distribution due to the improved road network. A comparison between the 2010 and the 2020 distribution shows that the probability of long delays decreases, whereas the probability of shorter delays increases for the 2020 network. This is the direct consequence of the assumption that travel time variability is positively related to the mean delay.

**Fig. 3:**
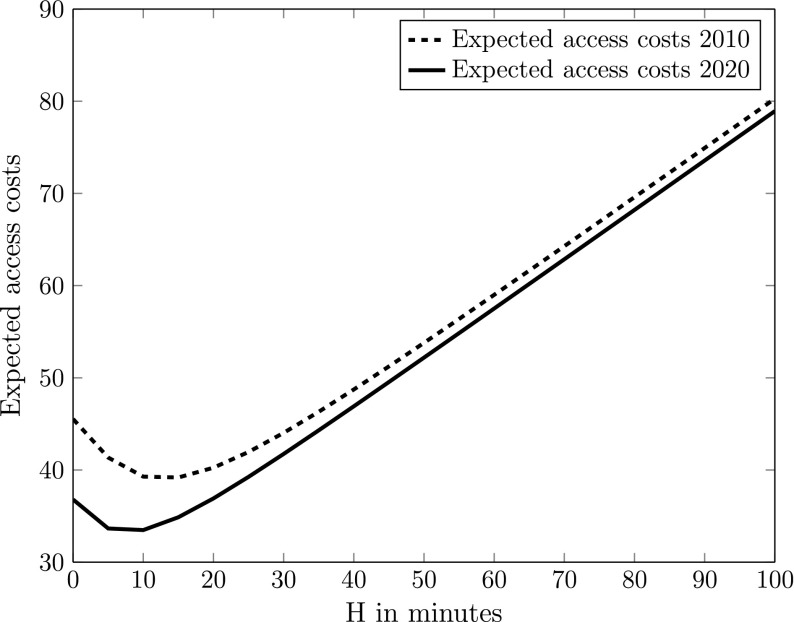
Expected access costs 2010 and 2020 for a business traveller (The Hague–Amsterdam Schiphol Airport, morning peak)

Because the travel time distribution changes between 2010 and 2020, the behavioural response of the travellers changes as well. Since the mean delays and the delay variabilities are lower in 2020, the model predicts that the traveller will depart later from home in 2020, resulting in a reduced optimal safety margin *H*. This can be seen if we plot the expected access cost function (Eq. 5), as a function of the safety margin *H* (with a step-size of 5 min). We use the willingness to pay values for business travellers as given in Table 1 (Fig. 3).

For 2010, the lowest expected access cost is achieved at a safety margin of 15 min, whereas for 2020 the optimal safety margin is equal to 10 min. Also, the corresponding optimal expected access costs decrease because of the improvement in the mean delay and the travel time reliability. For a given safety margin, the expected access costs for 2020 are always lower than the expected access costs for 2010.

#### Results for the entire road network

Next, we present the aggregate results (Table 3). The analysis of the previous section is repeated for all 1379 OD-pairs to obtain monetary estimates for the improvements in mean delays and travel time reliability due to road network investments in the Netherlands between 2010 and 2020. Tables 4 and 5 show the results for business and non-business travel respectively.

The results demonstrate that the largest cost improvements are realized during the morning peak. This is because congestion is most severe during this time of the day, and therefore the corresponding marginal reduction in costs is substantial. Surprisingly, the average travel time cost savings are largest for non-business travellers. It can be shown that this is due to the fact that non-business travellers travel relatively more often on links with larger improvements in mean delays.

The average absolute improvement in access costs per trip is not large (€ 1.86 for business and € 1.7 for non-business travellers), especially when compared to the spendings on airline tickets. This means that the accessibility of Schiphol is not expected to improve substantially due to the planned road network investments for 2020. The reason for these results is straightforward: as Table 3 shows, most travellers travel outside the morning peak to Amsterdam Schiphol Airport. The potential and willingness of policy makers to improve mean travel times and reliability during periods with little recurrent congestion is limited. Our results are thus different from the (more common) models that derive the benefits of network reliability for commuters: there most of the travellers will travel during peak hours, resulting in higher benefits from improvements in reliability.

**Table 4:** Cost improvement in € per trip for business travellers (B) during morning peak (MP), off-peak (OP) and evening peak (EP)

	BMP	BOP	BEP	Average	Percent
Total cost savings per trip	6.72	0.69	1.92	1.86	100
Travel time cost savings per trip	3.79	0.36	1.26	1.06	56.72
Travel time variability cost saving per trip	2.93	0.33	0.67	0.81	43.28

**Table 5:** Cost improvement in € per trip for non-business (NB) travellers during morning peak (MP), off-peak (OP) and evening peak (EP)

	NBMP	NBOP	NBEP	Average	Percent
Total cost savings per trip	6.91	0.7	1.61	1.7	100
Travel time cost savings per trip	4.18	0.39	1.11	1.02	59.83
Travel time variability cost saving per trip	2.73	0.31	0.5	0.68	40.17

However, the relative contribution of travel time variability improvements in total cost improvements is still substantial. Between 40% (non-business travellers) and 43% (business travellers) of the total cost savings are due to the reduction of access travel time variability. This implies that passengers’ benefits of improvements in the road network are underestimated to a substantial degree if reliability effects are ignored.

The improvements in network reliability also result in a slightly lower number of travellers who miss their flight. Tables 6 and 7 show the probabilities of missing a flight for business and non-business travellers for the years 2010 and 2020. The probability of missing a flight is highest during the morning peak with average values of 0.57% for business and 0.08% for non-business in 2010. This is because travel time variability is highest during the morning peak. Note that all probabilities are in the range 0–1.8%, which is considered reasonable when compared to real world data. It is also within the range used in the stated choice experiment of Koster et al. (2011). For all time periods the probability of missing a flight drops substantially when the network of 2020 is implemented, as a consequence of its inherent reliability improvements for both business and non-business travellers.

**Table 6:** Percentage of flights missed (2010) by business (B) and non-business (NB) travellers during the three time periods (MP, OP, EP)

	BMP	BOP	BEP	NBMP	NBOP	NBEP
Minimum	0	0	0	0	0	0
Median	0.5469	0.0117	0.0509	0.347	0.0105	0.0245
Average	0.5687	0.0546	0.073	0.4009	0.0517	0.0358
Maximum	1.7659	1.5336	0.4422	1.5315	1.1797	0.2531

**Table 7:** Percentage of flights missed (2020) by business (B) and non-business (NB) travellers during the three time periods (MP, OP, EP)

	BMP	BOP	BEP	NBMP	NBOP	NBEP
Minimum	0	0	0	0	0	0
Median	0.0336	0.001	0.0202	0.0145	0.0008	0.011
Average	0.0812	0.0084	0.0347	0.0459	0.0113	0.0192
Maximum	0.8241	1.4056	0.2359	0.564	1.0778	0.1186

We also benchmarked the results of our model against a simpler approach, which corresponds to ’Method 1’ of De Jong and Bliemer (2015), and is sometimes also referred to as mean-variance or reliability-ratio approach (named after the reliability ratio (RR), which is defined as the ratio of the value of reliability and the value of travel time $\alpha$).

The expected access costs in this simplified approach are given by:

7 $$\begin{aligned} E(C)=\alpha (T_{f}+\mu )+RR\alpha \sigma , \end{aligned}$$

Unlike the cost function used in the above analysis (Eq. 5), it does not consider schedule delays (explicitly), and it also lacks a discrete penalty for missing the flight. Moreover, the reliability ratio approach requires the standardized travel time distributions to be the same before and after the network change.

By setting equal the expected access costs of the simpler method (Eq. 7) and the expected access costs from the method developed in this paper (Eq. 5),^13^ and then solving for RR, we can derive the implied reliability ratio (IRR), which is the RR that produces exactly the same result as our scheduling method. It is given by:

8 $$\begin{aligned} IRR=\frac{E(C(H^{*}))-\alpha \left( T_f+\mu \right) }{\sigma VOT} \end{aligned}$$

**Fig. 4:**
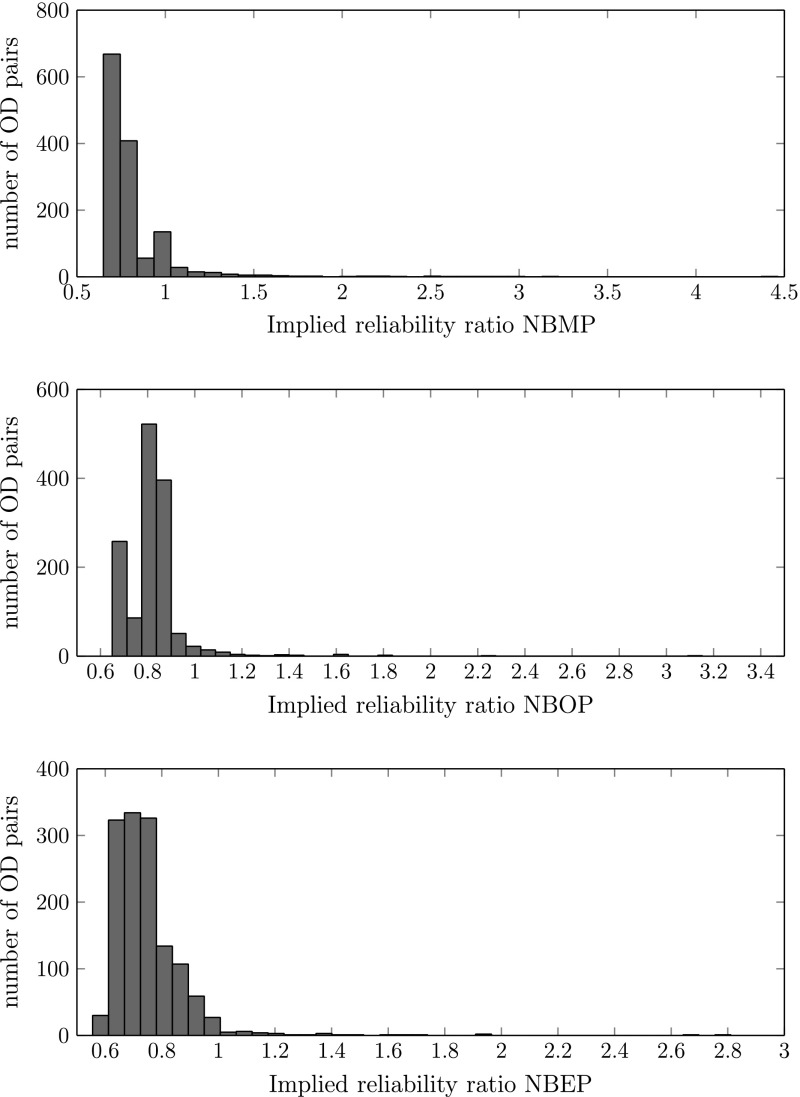
Implied reliability ratios for non-business (NB) travellers during morning peak (MP), off-peak (OP) and evening peak (EP), year 2020

We have computed the IRR for each of the 1378 OD pairs, each day period, each travel purpose, and for the 2010 and the 2020 network. For both, business and non-business travellers, we find that the IRRs vary substantially across OD-pairs, time periods and years. As an illustration, histograms of the IRRs for non-business travellers for the year 2020 and the three time periods are shown in Fig. 4.^14^ Our results suggest that valuable information is lost if the reliability ratio approach is applied.^15^ The implications of these results are especially strong if one is interested in analysing the costs of travel time variability on specific road stretches: there, the results of both methods differ substantially, whereas the variations tend to average out if larger parts of the network are analysed.^16^

## Conclusions

We developed a practical method to estimate the benefits of improvements in road net-work reliability. It allows for the estimation of reliability benefits without requiring the use of a full blown dynamic network simulation model, while still capturing the essential behavioural response of drivers to travel time variability. The model is based on a standard scheduling model for departure time choice, and uses as inputs the travel time estimates of a standard static transport model and an empirical (OD-pair-specific) function that describes the relation between mean delays and travel time variability. Moreover, we assume that delays are log-normally distributed.

We applied the model to air passengers travelling to the Amsterdam Schiphol airport in order to catch a flight, hence a situation where access travel time reliability is crucial. We compared the road network in the Netherlands in 2010 to the planned network in 2020, under the assumption that travel demand is inelastic. For the time period between 2010 and 2020 various improvements of the network shall take place, leading to shorter average travel times as well an improved reliability.

We found that the average improvements in access travel costs are fairly small in absolute terms, mainly because most passengers travel to the airport outside the peak hours. However, the relative contribution of reliability benefits was substantial: our results showed that the total benefits from infrastructure improvements are about 70% higher when benefits due to better reliability are taken into account in addition to the savings in mean travel time alone. This number is quite high compared to the estimates reported in earlier studies, which tend to be in the range of 10–25% (e.g. Fosgerau et al. 2008; Eliasson 2006; Peer et al. 2012). A main reason is that our application concerns travel to the airport, a situation in which on-time arrival is crucial, which is reflected in the scheduling preferences we apply. However, because we assumed that overall demand is inelastic, our estimate of the total benefits might still be an underestimate of the real effect because we ignored the additional consumer surplus stemming from new air travellers entering the road network because of lower generalised costs.

We compared our results with the results of a simpler (so-called mean-variance or reliability-ratio) approach, which assumes a linear relationship between the costs of travel time variability and the standard deviation of the delays. We found that the reliability ratio (i.e. the ratio of the value of reliability and the value of travel time) that implies a cost equivalence between our approach and the more simplified one, differs substantially across OD-pairs (but also across the two network settings, the time of the day and the travel purpose). From that we concluded that especially when looking at smaller parts of the network, where such heterogeneity is likely to exist, our approach is able to capture a more realistic picture.

One limitation of our analysis is the assumption that flights always depart on time. If flights are delayed, the probability of missing a flight may be overestimated in our analysis. Furthermore, our assumption that delays on the road and in the air are independent from each other may not always hold in reality. For instance, adverse weather conditions may cause delays for both car and air travel. We leave this interplay of access delays and flight delays as a topic for further study.

Future research may also focus on obtaining more detailed estimates of the benefits of improvements in network reliability. We expect that our model could be made more accurate by employing a more sophisticated method to estimate the standard deviation of delays, for example by incorporating road characteristics. Second, one could allow for more flexibility in the shape of the travel time distributions as the log-normal distribution might not approximate the true distribution of travel times well enough. These improvements could be easily accommodated within the framework of our model, and could lead to more precise estimates of the travel time distribution and the corresponding travel costs.

## Footnotes

1 Our method uses the mean delays on links and origin–destination-paths that are predicted by the equilibrium assignment (Wardrop equilibrium) module of a standard transport model. While the transport model does account for the effects of traffic congestion in the road network, it does not include any feedback of the variability effect back to the route choice of the car drivers or to other choices represented within the transport demand model.

2 Note that travel time variability itself (i.e. its extent) is still defined in terms of the standard deviation of the travel time distribution.

3
Noland and Small (1995) were the first to develop this idea by employing an expected utility model of departure time choice based on the scheduling model of Vickrey (1969) and Small (1982). Noland and Small (1995) assumed that delays follow a uniform or exponential distribution. Their model was later extended by Fosgerau et al. (2010) for general travel time distributions.

4 Note that the cost function used here can be adapted easily to fit a more generic setting.

5 First note that $F_{logn}(D)=\frac{1}{2}Erfc\left[ \frac{\tau -\ln \left[ D\right] }{\sqrt{2}\kappa }\right]$, where *Erfc* is the complementary error function. Then we use that $\int \nolimits_{0}^{H}Df_{logn}(D)dD=\frac{1}{2}\mu Erfc\left[ \frac{\tau -\ln \left[ H\right] +\kappa ^2}{\sqrt{2}\kappa }\right]$. Substituting $D=H/\exp (\kappa ^2)$ in $F_{logn}(D)$ gives the desired result.

6
Bates et al. (2001) show that for any travel time distribution it must be true that $\mathbb {E}(SDE;H)-\mathbb {E}(SDL;H)=(\mu -H)$, which is confirmed by Eqs. 2 and 3.

7 The expected access cost function can be easily programmed in software packages such as Matlab.

8 Note that we will apply the highway-data-based estimates of Kouwenhoven and Warffemius (2015) to door-to-door trips, which naturally also include travel along urban and provincial roads. The main reason is that so far little empirical evidence exists concerning the relationship between mean delay and the standard deviation for roads other than motorways. Kouwenhoven and Warffemius (2015) provide some evidence, however, based on travel time data from only 40 routes along urban and provincial roads. Possibly due to the low number of observations, they find various coefficients to be insignificant, and additionally a worse model fit than for the highway data.

9 The standard deviation is computed for each 15-min interval between 00:00 and 23:00 across all working days in 2012.

10 Note that for small values of the mean delay, the predicted standard deviation may become negative. It is set to 0 for these cases.

11 We will apply the coefficients for the mid-day period to all periods outside the morning and evening peak (“off-peak”).

12 Under the assumption of 320 working days per year.

13 Note that the costs associated with travel time are equal across both methods.

14 More detailed results of this comparison are available from the authors upon request.

15
Kouwenhoven et al. (2014) suggest for the Netherlands an RR of 1.1 for business travellers and of 0.6 for non-business travellers.

16 In fact, Börjesson et al. (2012) showed that the assumptions under which the scheduling and reduced-form models are theoretically equivalent, may not hold empirically. This was another motivation for us to use the scheduling model at first instance.

## References

[CR1] Bakker, D., Mijjer, P., Daly, A., Vrolijk, P.: Prediction and evaluation of the effects of traffic management measures on congestion and vehicle queues. PTRC European Transport Forum, Warwick (1994)

[CR2] Bates J, Polak J, Jones P, Cook A (2001). The valuation of reliability for personal travel. Transp. Res. E Logist. Transp. Rev..

[CR3] Börjesson M, Eliasson J, Franklin JP (2012). Valuations of travel time variability in scheduling versus mean-variance models. Transp. Res. B Methodol..

[CR4] De Jong GC, Bliemer MC (2015). On including travel time reliability of road traffic in appraisal. Transp. Res. A Policy Pract..

[CR5] Eliasson, J.: Forecasting travel time variability. In: Proceedings of the European Transport Conference (2006)

[CR6] Emam E, Ai-Deek H (2006). Using real-life dual-loop detector data to develop new methodology for estimating freeway travel time reliability. Transp. Res. Rec. J. Transp. Res. Board.

[CR7] Fosgerau M, Karlström A (2010). The value of reliability. Transp. Res. B Methodol..

[CR8] Fosgerau, M., Hjorth, K., Brems, C.R., Fukuda, D.: Travel time variability: definition and valuation. Technical report, Technical University of Denmark, Transport (2008)

[CR9] Fosgerau M, Hjorth K, Lyk-Jensen SV (2010). Between-mode-differences in the value of travel time: self-selection or strategic behaviour?. Transp. Res. D Transp. Environ..

[CR10] Gaver DP (1968). Headstart strategies for combating congestion. Transp. Sci..

[CR11] Gunn H (1994). The Netherlands National Model: a review of seven years of application. Int. Trans. Oper. Res..

[CR12] Hall R (1983). Travel outcome and performance: the effect of uncertainty on accessibility. Transp. Res. B Methodol..

[CR13] Knight T (1974). An approach to the evaluation of changes in travel unreliability: a safety margin hypothesis. Transportation.

[CR14] Kouwenhoven, M., Warffemius, P.: Quantifying the socio-economic benefits of transport. Discussion paper, International Transport Forum, Paris (2015)

[CR15] Koster P, Kroes E, Verhoef E (2011). Travel time variability and airport accessibility. Transp. Res. B Methodol..

[CR16] Kouwenhoven M, de Jong GC, Koster P, van den Berg VA, Verhoef ET, Bates J, Warffemius PM (2014). New values of time and reliability in passenger transport in The Netherlands. Res. Transp. Econ..

[CR17] Noland R, Small K (1995). Travel-time uncertainty, departure time choice, and the cost of morning commutes. Transp. Res. Rec..

[CR18] Peer S, Koopmans CC, Verhoef ET (2012). Prediction of travel time variability for cost–benefit analysis. Transp. Res. A Policy Pract..

[CR19] Rakha H, El-Shawarby I, Arafeh M (2010). Trip travel-time reliability: issues and proposed solutions. J. Intell. Transp..

[CR20] Small KA (1982). The scheduling of consumer activities: work trips. Am. Econ. Rev..

[CR21] Thomson J (1968). The value of traffic management. J. Transp. Econ. Policy.

[CR22] Vickrey WS (1969). Congestion theory and transport investment. Am. Econ. Rev..

